# Urinary Markers in Bladder Cancer: An Update

**DOI:** 10.3389/fonc.2018.00362

**Published:** 2018-09-07

**Authors:** Giorgio Santoni, Maria B. Morelli, Consuelo Amantini, Nicola Battelli

**Affiliations:** ^1^Immunopathology Laboratory, School of Pharmacy, University of Camerino, Camerino, Italy; ^2^Immunopathology Laboratory, School of Biosciences, Biotechnology and Veterinary Medicine, University of Camerino, Camerino, Italy; ^3^Oncology Unit, Macerata Hospital, Macerata, Italy

**Keywords:** urinary biomarkers, bladder cancer, liquid biopsy, microRNA, exosomes

## Abstract

Bladder cancer (BC) is ones of the most common cancer worldwide. It is classified in muscle invasive (MIBC) and muscle non-invasive (NMIBC) BC. NMIBCs frequently recur and progress to MIBCs with a reduced survival rate and frequent distant metastasis. BC detection require unpleasant and expensive cystoscopy and biopsy, which are often accompanied by several adverse effects. Thus, there is an urgent need to develop novel diagnostic methods for initial detection and surveillance in both MIBCs and NMIBCs. Multiple urine-based tests approved by FDA for BC detection and surveillance are commercially available. However, at present, sensitivity, specificity and diagnostic accuracy of these urine-based assays are still suboptimal and, in the attend to improve them, novel molecular markers as well as multiple-assays must to be translated in clinic. Now there are growing evidence toward the use of minimally invasive “liquid biopsy” to identify biomarkers in urologic malignancy. DNA- and RNA-based markers in body fluids such as blood and urine are promising potential markers in diagnostic, prognostic, predictive and monitoring urological malignancies. Thus, circulating cell-free DNA, DNA methylation and mutations, circulating tumor cells, miRNA, IncRNA and mRNAs, cell-free proteins and peptides, and exosomes have been assessed in urine specimens. However, proteomic and genomic data must to be validated in well-designed multicenter clinical studies, before to be employed in clinic oncology.

## Introduction

Bladder cancer (BC) represents the 9th and 4th most common cancer worldwide and in men in the USA, respectively (1, 2). Its main histological type is urothelial carcinoma (UC). About 70–80% of BC is diagnosed as non-muscle invasive BC (NMIBC) and 20–30% as muscle invasive (MIBC). Because 10–30% of patients with NMIBC progress to invasive disease (3–8), early diagnosis and early detection of recurrence are very important. BC diagnosis requires cystoscopy and biopsy, which are unpleasant and costly procedures (9). It is necessary to develop new diagnostic methods less invasive and expensive for BC diagnosis and surveillance. The Food and Drug Administration (FDA) has approved the use of multiple urine-based tests that are commercially available. However, none of these tests has been routinely used and incorporated in the American Urological Association or in the European Association of Urology clinical guidelines for BC treatment (10). In this mini-review we discuss the clinical implementation by the use of novel molecular approaches and liquid biopsy in BC.

At present, the gold standard methods for BC diagnosis are urine cytology and cystoscopy. Cytopathology of urine specimens is the widely used non-invasive test for detection and surveillance of BC (11–13). Cytology is very specific (about 86%), but it is low sensitive (48%) limiting its use in low-grade BC (14–16). Diagnostic accuracy of urinary cytology is subjective, depending on cytopathologist expertise (17). Thus, new molecular-based urinary tests for reducing or substituting, the endoscopy frequency in BC recurrence patients, are required (18, 19).

Advanced technology utilizes patients' urine as samples instead of primary BC tissues to identify novel predictive biomarkers. At present, the major problem is to translate the extensive proteomic and genomic data in clinical practice and to validate the expression of these biomarkers in well-designed multicenter clinical studies (20).

## Proteomic and peptidomic analysis

Proteomic analyses have opened a new horizon for cancer biomarker discovery (21). At present, seven tests are available: FDA approved six on seven of these tests, and the last one is in agree with the Clinical Laboratory Improvement Act standards. NMP22, NMP22 BladderChek, and UroVysion have FDA approval for BC diagnosis and surveillance; immunocytology (uCyt+), BTA-TRAK, and BTA-STAT have been approved only for surveillance (22–26).

In order to improve sensitivity, specificity and diagnostic accuracy in BC diagnosis, novel protein markers, waiting to be approved, are used experimentally. BCLA-1 and BCLA-4 are nuclear matrix proteins specifically targeting BC tissues, with no interference with infection, smoking, catheterization or cystitis (27). In patients with hematuria, aurora A kinase (AURKA) discriminates between low-grade BC vs. normal patients (28). The Aura Tek FDP Test™ in urine can detect BC recurrence (29). The activated leukocyte cell adhesion molecule (ALCAM), a cell adhesion molecule (30), positively correlates with tumor stage and overall survival (OS), after adjusting for patients, clinical features and Bacillus Calmette-Guerin treatment (31). Nicotinamide N-methyltransferase is high in BC patients and correlate with histological grade (32). Apurinic/apyrimidinic endonuclease 1/redox factor-1 (APE/Ref-1) levels are higher in BC, respect to non-BC, and correlate with tumor grade and stage; moreover it is high also in patients with recurrence history of BC (33). The cytokeratin-20 (CK20) urine RT-PCR assay shows 78–87% sensitivity and 56–80% specificity for urothelial BC detection, with improved diagnostic accuracy in tumor progression (34) but it has poor performance for low-grade tumors. Higher levels of CK8 and CK18 was detected in the urine by UBC Rapid test in high- vs low-grade BC (35).

As multiple markers for BC detection, increased urinary levels of apolipoprotein A1, A2, B, C2, C3, *E* (APOA1, APOA2, APOB, APOC2, APOC3, APOE) were found in BC relative to healthy controls (36, 37). A signature of 4 urinary fragments of uromodulin, collagen α-1 (I), collagen α-1 (III), and membrane-associated progesterone receptor component 1 seems to discriminate MIBCs from NMIBCs (38). Other panel employs IL-8, MMP-9/10, ANG, APOE, SDC-1, α1AT, PAI-1, VEGFA, and CA9 to diagnose BC starting from urine samples (39). The advantage of these multi-urinary protein biomarkers was evident in high- and low-grade and high- and low-stage disease (39). The combination of urinary markers such as midkine (MDK) and synuclein G or MDK, ZAG2 and CEACAM1 (40), angiogenin and clusterin (41) evaluated by immunoassay and urine cytology increases the sensitivity and specificity in NMIBC diagnosis (40). Increased CK20 and Insulin Like Growth Factor II (IGFII) levels were detected in the urine sediments of NMIBC patients compared to controls (42). Increased levels of urinary HAI-1 and Epcam evaluated by ELISA, are prognostic biomarkers in high-risk NMIBC patients (43). Urinary survivin evaluated by chemiluminescence enzyme immunoassay correlates with tumor stage, lymph node and distant metastases and represents a potential marker for preliminary BC diagnosis (44). Snail overexpression represents an independent prognostic factor for tumor recurrence in NMIBC (45). Finally, specific glycoproteins were identified by glycan-affinity glycoproteomics nanoplatforms in the urine of low- and high-grade NMIBC; among these, increased urinary CD44 levels were evidenced in high-grade MIBC (46).

Urinary metabolomics signature could also be useful in early BC. By ultra-performance liquid chromatography time and mass spectrometry, imidazole-acetic acid was evidenced in BC (47). Moreover, acid trehalose, nicotinuric acid, AspAspGlyTrp peptide were upregulated; inosinic acid, ureidosuccinic acid and GlyCysAlaLys peptide were downregulated in BC, but not in normal cohort (48). A metabolite panel with indolylacryloylglycine, N2-galacturonyl-L-lysine and aspartyl-glutamate permits to discriminate high- vs. low-grade BC (49). In addition, the alteration of phenylalanine, arginine, proline and tryptophan metabolisms was evidenced by UPLC-MS in NMBIC (50).

## Circulating tumor and cell-free DNA

Tumors release DNA fragments into circulation, called circulating tumor DNA (ctDNA) containing tumor-specific mutations, variations of copy number and alterations in DNA methylation status. This ctDNA reflects the heterogeneity of tumor subclones. In BC patients, ctDNA is detectable in over 70% of urine samples (51) and it allows to discriminate between BC patients and control subjects (52). CtDNA measures about 180 and 200 base pairs. It is easily accessible, but it is rapidly cleared from circulation following systemic therapy (53). PCR-based approaches, and more recently, digital-PCR and genome sequencing, represent the methods of choice for cell-free DNA (cfDNA) analysis.

### DNA methylation

The methylation status of tumor-related genes represents a very important epigenetic alteration affecting cancer initiation and progression. Hyper- and hypo-methylated regions are identified in BC and in premalignant lesions. Alterations in DNA methylation status are chemically stable, develop early during tumorigenesis and can be assessed in circulating cfDNA fragments and in cells shed into the urine (54). A significant prevalence of methylated genes, for example APC and cyclin D2, was found in the urine from malignant vs. benign cases (55). Hyper-methylation in GSTP1 and RARβ2 and APC genes has been identified in the urine from BC patients (56). The evaluation of Twist Family BHLH Transcription Factor 1 (TWIST1) and NID2 genes methylation status in urine permits to differentiate primary BC patients from controls with 90% sensitivity and 93% specificity (57). In addition, the evaluation of the methylation status of NID2 and TWIST1 or CFTR, SALL3 and TWIST1 genes in urinary cells in combination with cytology, has been found to increase sensitivity and high negative predictive value in BC patients (58, 59). The analysis of 1,370 loci specific DNA methylation patterns seem to permit to distinguish NMIBC from MIBC (60). Sun and coworkers demonstrated higher recurrence predictivity than urine cytology and cystoscopy (80 vs. 35 vs. 15%) by using SOX-1, IRAK3, and Li-MET genes methylation status from urine sediments of BC patients (54). POU4F2 and PCDH17 methylation levels in urine distinguish BC from normal controls with 90% sensitivity and 94% specificity (61). Promoter hyper-methylation of HS3ST2, SEPTIN9 and SLIT2 genes combined with FGFR3 mutation showed 97.6% sensitivity and 84.8% specificity for diagnosis, surveillance and risk stratification in low- or high-risk NMIBC patients (62). Finally, the methylation status of p14ARF, p16INK4A, RASSF1A, DAPK, and APC tumor suppressor genes has been found to correlate with BC grade and stage (63).

Altogether, although promising results were obtained, accuracy of urinary methylated DNA is variable and results still await validation studies and complementary markers for clinical implementation (64, 65). In this regard, the recent introduction of the methylation-sensitive High Resolution Melting and Methylated CpG Island Recovery methods could further increases the sensitivity for the detection of methylome in BC urine (Table 1) (72, 73).

**Table 1:** Urinary tumor-derived DNAs as biomarkers in BCs.

**Urinary tumor-derived DNA**	**Gene**	**Application**	**References**
CfDNA	TERT and FGFR3	Recurrence	(66, 67)
	TERT, FGFR3/OTX1	BC diagnosis	(68)
	FGFR3 and PIK3CA	Progression/metastasis	(69)
Histone modifications	HAK20me3	Poor survival	(70)
	H3K27me3	Poor survival	(71)
DNA methylation status	GSTP1 and RARb2 and APC	BC diagnosis	(56)
	TWIST1 and NID2	BC diagnosis	(57)
	SOX-1, IRAK3, and Li-MET	Recurrence	(54)
	POU4F2 and PCDH17	BC diagnosis	(61)
	HS3ST2, SEPTIN9, SLIT2/FGFR3	surveillance, low vs. high risk	(62)
	NID2 and TWIST1	BC diagnosis	(58)
	CFTR, SALL3/TWIST1	BC diagnosis	(59)
	p14ARF, p16INK4A, RASSF1A	BC grade and stage	(63)
	DAPK and APC		

*BC, Bladder cancer; CfDNA, circulating-free DNA*.

### cfDNA, mutation and microsatellite alterations

Since tumor-derived DNA can be released into circulation and mutations in cfDNA can be detected in various biological fluids, their use as non-invasive cancer biomarkers has been proposed. Urinary TERT promoter mutations, that occur early in urothelial neoplasia, FGFR3 mutation and telomere length correlate with high-risk BC recurrence (66, 67). TERT, evaluated by telomeric repeat amplification protocol, in combination with FGF3 and OTX1 shows high sensitivity in NMIBCs as well as in pT1 tumors and in high-grade BC (68). In addition, increased FGFR3 and PIK3CA mutated DNA levels in urine has been found to be indicative of progression and metastasis in NMIBC (69). Microsatellite analysis in circulating DNA of BC patients targets highly polymorphic, short tandem repeats. Loss of heterozygosity (LOH) analysis is more sensitive than urine cytology (97 vs. 79%), particularly for low-grade BC diagnosis. It also significantly improves the detection of low-grade and low-stage BC, with 95% sensitivity for G1-G2 grades and 100% for pTis and pTa tumors (Table 1) (74).

### Histone tail modifications

The levels of histone methylation are lower in advanced tumors respect to controls and correlated to poor survival. Thus, increased levels of HAK20me3 were evidenced in a MIBC subset (70); furthermore high H3K27me3 levels correlate with worse survival after cystectomy in pT1-3 and pN- BC patients (71). H2AFX1 gene methylation was detected in paraffin-embedded BC and its expression correlated with increased recurrence rates (Table 1) (75).

## Urinary tumor RNA

Several RNA classes, messenger RNAs (mRNAs), microRNAs (miRs) and long non-coding RNAs (lncRNAs), have been recognized as potential non-invasive cancer biomarkers (76). Altered levels of circulating RNAs in cancer, which returned to normal following surgery have been reported (77), suggesting release of RNA molecules from tumors.

### miRNAs (miRNAs)

miRNAs are short (21–23 nucleotides length) non-coding RNAs regulating gene expression by pairing to the 3′untranslated region (UTR) of their target mRNA. Several miRNAs have been found to play an important role in tumorigenesis, progression and metastasis of cancer cells (78, 79). Urine seems to be a good source for miRNA detection for its content of cell-free nucleic acid in supernatant or sediments (80). However, the diagnostic significance in the detection of miRs in urine as respect to blood of BC patients is controversial (81). MiR-126 urinary levels were found to be enhanced in BC compared to healthy controls (82). Urine miR-146a-5p is significantly increased in high-grade BC (77). Low miR-200c expression correlates with tumor progression in NMIBCs (83). Chen et al. detected 74 miRNAs, of which 33 upregulated and 41 downregulated in BC compared to healthy patients (84). The most interesting are let-7miR, mir-1268, miR-196a, miR-1, miR-100, miR-101, and miR-143 (84). MiR-200 was identified as epithelial–mesenchymal transition regulator in BC cells by targeting Zinc Finger E-Box Binding Homeobox 1 (ZEB1), ZEB2 and Epidermal growth factor receptor (EGFR) (85). Some miRNAs have been associated with hemolysis including miR-451a, miR-16, miR-486-5p, and miR-92a (86). Eissa et al. by screening BC patients with negative cystoscopy, identified miR-96 and miR-210 in BC (87). Sapre et al., by using a panel of 12 miRNA, reduced the cystoscopy rates by 30% by increasing sensitivity and specificity (88). MiR-125b, miR-30b, miR-204, miR-99a, and miR-532-3p were downregulated in BC patient's urine supernatant, with miR-125 levels (95.7% specificity, 59.3% sensitivity) (89). MiR-9, miR-182 and miR-200b correlated with MIBC aggressiveness, recurrence-free and OS (90). MiR-145 distinguishes NMIBCs from non-BCs (91). MiR-144-5p inhibited BC proliferation, affecting CCNE1, CCNE2, CDC25A, PKMYT1 target genes (92). Cell-free urinary miR-99a and miRNA-125b were found to be downregulated in the urine supernatants of BC patients (sensitivity 86.7%; specificity 81.1%) (93). Urinary levels of miR-618 and miR-1255b-5p in MIBC patients were increased in comparison to controls (94). Multiple miRNA assay shows higher diagnostic performance than single RNA assay (95). By whole genome analysis increased miR-31-5p, miR-191-5p and miR-93-5p levels were identified in the urine of BC patients as compared to controls (96).

Recently, a miRNA profile, identified in urine by next-generation sequencing (NGS) analysis, has been capable to stratify different BC subtypes (97). In NMIBC G1/G2 patients a miR-205-5p upregulation compared to controls was observed. Among NMIBC G3, upregulation of miR-21-5p, miR-106b-3p, mir-486-5p, miR-151a-3p, miR-200c-3p, miR-185-5p, miR-185-5p and miR-224-5p and downregulation of miR-30c-2-5p and miR-10b-5p were observed. In MIBCs, miR-205-5p, miR-451a, miR-25-3p and miR-7-1-5p were upregulated, while miR-30a-5p was downregulated compared to controls (97). The application of NGS have increased the diagnostic accuracy. However results obtained in NGS were only partially overlapping with that obtained by qRT-PCR (98) (Table 2).

**Table 2:** Urinary tumor-derived RNAs as biomarkers in BCs.

**Urinary tumor-derived RNAs**	**RNA/Protein**	**Application**	**References**
mRNA	CK20, IGF-II	BC diagnosis	(42)
	ABL1, CRH, IGF2, UPK1B and ANXA10	BC diagnosis	(78)
	NDRG2	Tumor grade and stage	(99)
miRNA	miR-146a	BC diagnosis	(77)
	miR-126	BC diagnosis	(82)
	miR-200c	Tumor progression	(83)
	let-7,miR-1268,−196a,−1,−101,−143	BC diagnosis	(84)
	miR-451a,−16,−486,−92a	Hemolysis	(86)
	miR-96,−210	BC diagnosis	(87)
	miR-125b,−30b,−204a,−99a,−532	BC diagnosis	(89)
	miR-9,−182,−200b	aggressiveness, recurrence	(90)
	miR-145	BC diagnosis	(91)
	miR-99a,−125b	BC diagnosis	(93)
	miR-618,−1255b	BC diagnosis	(94)
	miR-21,−106b,−486,−151a,−200c	NMIBC diagnosis	(97)
	−185,−224, 30c-2,−10b		
	miR-205,−451a,−25,−7-1,−30a	MIBC diagnosis	(97)
	miR-31,−191,−93	BC diagnosis	(96)
miRNA/EVs and Exosomes	miR-375,−146a	BC diagnosis	(100)
miRNA/tRF	miR720/3007a	BC diagnosis	(101)
IncRNA	uc004cox.4	Recurrence	(102)
IncRNA/exosomes	HOX-AS, ANRIL, and linc-RoR	BC diagnosis	(103, 104)

*BC, Bladder cancer; NMIBC, muscle non-invasive BC; MIBC, muscle invasive; mRNA, messenger RNA; miR, microRNA; EVs, extracellular vesicles; tRF, transfer RNA fragments; IncRNA, Long non-coding RNA*.

### Long non coding RNAs (IncRNAs)

Long non coding RNAs (lncRNAs) regulate gene expression or epigenetic levels. Several findings show lncRNA changes in cancers suggesting a role in the promotion of tumor development and progression (105, 106). The use of lncRNAs as non-invasive BC marker has recently interested (107). Circulating urothelial carcinoma antigen 1 (UCA1) levels in urinary sediments represents a potential diagnostic marker for UC, with 81% sensitivity and 92% specificity (108). Du et al. describe high uc004cox.4 IncRNA level association with poor recurrence-free survival in NMIBCs (102). The retrotrasposome, long interspaced element-1 (LINE-1) has been found to be hypo-methylated and its expression was associated with long recurrence-free and tumor specific survival in BC (109) (Table 2).

### Messenger RNAs (mRNAs)

Circulating messenger RNAs (mRNAs) were detected in cancer patients, although the majority of circulating mRNAs are degraded by RNases (110). Given their role in intracellular protein translation, their presence reflects the status of intracellular processes and they are potential cancer biomarkers. Urine Ubiquitin Conjugating Enzyme E2 C (UBE2C) mRNA levels were higher in BC patients, compared to normal and hematuria specimens (111). The expression of isoleucine glutamine motif-containing GTAase-activating proteins (IQGAP3) mRNA in urine was found higher in BC than in controls (112). Further analysis of IQGAP3, with respect to tumor invasiveness and grade also yielded a high diagnostic accuracy, suggesting that IQGAP3 can be used to discriminate BC from non-BC patients with hematuria (112).

In regard to mRNAs extracted by exfoliated urinary cells, the Xpert BC Monitor measuring ABL1, corticotropin releasing hormone (CRH), IGF2, uroplakin 1B (UPK1B), annexin A10 (ANXA10) mRNAs by RT-PCR, increased the overall sensitivity over urinary cytology in low-grade and pTa disease (113).

In addition, the presence of carbonic anhydrase 9 (CAIX) splice variant mRNA in the urine, increased the diagnostic performance for BC (90% sensitivity and 72% specificity) (114). The downregulation of N-Myc downstream-regulated gene 2 (NDRG2) mRNA levels in the urine of BC patients correlated with tumor grade and stage (99) (Table 2).

### Transfer RNA fragments (tRFs)

Elevated levels of transfer RNA fragments (tRF) are found in cancer (115). tRF are 14-32 base long single-stranded RNA derived from mature o precursor tRNA. They are grouped into 3 classes (tRF-1, −3, and −5) and, depending of their cleavage site within a mature RNA, they are further divided in 5 subclasses. The first identified tRF in NMIBCs was miR720/3007a (101) (Table 2).

## Extracellular vesicles (EVs) and exosomes

Extracellular Vesicles (EVs) enrichment was found in BC patient urine. EVs, analyzed by MS based proteomics, demonstrated specific protein and miRNAs pattern in BC patients (116). By using a microarray platform and RT-PCR analysis, miR-375, and miR146a have been found to specifically identify high-grade and low-grade BC, respectively (100). The application of nanowires anchored into a microfluidic substrate will enable the efficiency of EV collection, thus permitting to identify EV harboring miRNAs (117).

Exosomes are membrane vesicles secreted in nearly all body fluids at elevated levels in cancer patients relative to healthy subjects (118, 119). They realize intercellular communication through transferring distinct biologically active molecules (RNAs, DNA, and proteins), thus influencing the therapeutic responses. The HOX transcript antisense RNA (HOTAIR) together with other IncRNA, such as HOX-AS-2, ANRIL, and linc-RoR, were augmented in urinary exosomes from high-grade MIBC patients (103). Loss of HOTAIR expression in BC cells alters the expression of SNA1, TWIST1, ZEB1, ZO1, MMP-1, Laminin Subunit Beta 3 (LAMB3), and Laminin Subunit Gamma 2 (LAMC2) epithelial-to mesenchymal transition genes. Moreover, the tumor-associated calcium-signal transducer 2 (TACSTD2*)* was found in BC exosomes by proteomic analysis (104). EVs can also promote BC progression by delivering the protein EGF-like repeat and discoidin I-like domain-containing protein-3 (120).

Exosomes in urine also contain miRNAs, in particular miR-1224-3p, miR-135b, and miR15b; in particular, miR-126/miR-152 ratio correlated with positive BC diagnosis (121) (Table 2).

Although EVs and exosomes represent an interesting source of cancer biomarkers, the lack of accurate isolation and detection methods affects their utilization in practice. In the next future, the development of sensitive capture platforms for exosomes, likely increases their introduction into clinic.

## Urinary microbiome

Dysbiosis of urinary microbiome has been suggested to be involved in bladder tumorigenesis. Recently, Wu et al. by analyzing DNA extracted by urine pellets, observed specific enrichment of *Acinetobacter, Anaerococcus*, and *Sphingobacterium* in BC cohort as respect to controls (122). Moreover, the increase of *Herbaspirillum, Porphyrobacter*, and *Bacteroides* in high-risk BC patients suggested that these genera may represent new potential biomarkers (122).

## Conclusions and perspectives

We provide the state of art into the use of urinary biomarkers as tool to aid diagnosis of BC. Urine cytology, utilized for decades, shows poor sensitivity, particularly for low-grade tumors. The addition of immunoassay and FISH analysis has provided an additional diagnostic armamentarium to determine which patients may need further evaluation. At present, there are growing evidence toward the use of “Liquid Biopsy” to identify urinary biomarkers such as circulating cell-free DNA, DNA methylation, miRNA, cell-free proteins/peptides and exosomes, useful for discriminating NMIBC from MIBC (123). The potential introduction of “smart toilets” working with a more advanced “nano-sensor” able to detect RNA and proteins in urine is close to reality, more that we think (124). However, now in clinical reality, there is an urgent need to validate the recently discovered extensive proteomic and genomic, epigenomic, transcriptomic and metabolomic data as urinary biomarkers in well-designed multicenter clinical studies (125, 126).

## Author contributions

GS, MM conception and design. GS drafting the manuscript. CA and NB critical revision of the manuscript.

### Conflict of interest statement

The authors declare that the research was conducted in the absence of any commercial or financial relationships that could be construed as a potential conflict of interest.
